# Epistaxis and Hypertensive Emergency as the First Signs of Lupus Nephritis

**DOI:** 10.7759/cureus.81525

**Published:** 2025-03-31

**Authors:** Adham Mohsen, Husam El Sharu, Bryan K Dunn

**Affiliations:** 1 Nephrology/Critical Care, East Carolina University, Greenville, USA; 2 Internal Medicine, East Carolina University, Greenville, USA; 3 Pulmonary and Critical Care, East Carolina University Brody School of Medicine, Greenville, USA

**Keywords:** cyclophosphamide therapy, diffuse lupus nephritis, hypertensive emergency, microscopic hematuria, elderly onset lupus

## Abstract

Systemic lupus erythematosus is a multisystem autoimmune disease that predominantly affects young females. This case report describes a rare presentation of a hypertensive emergency as the initial manifestation of lupus nephritis (LN) in a 64-year-old female with normal serum creatinine at presentation. The patient initially presented with epistaxis and severe hypertension (221/127 mmHg). Further evaluation revealed non-nephrotic range proteinuria and microscopic hematuria. Autoimmune studies and renal biopsy confirmed diffuse proliferative LN (Class IV). Treatment with corticosteroids and cyclophosphamide yielded a favorable clinical response.

## Introduction

Systemic lupus erythematosus (SLE) is a chronic, multisystem autoimmune disease that predominantly affects the musculoskeletal, cardiovascular, and renal systems, with a higher prevalence in young females [1]. Diagnosis is typically informed by kidney biopsy findings of lupus nephritis (LN) or the classification criteria established by the European Alliance of Associations for Rheumatology (EULAR) and the American College of Rheumatology [2].

Hypertension is a common complication in SLE, affecting 14%-44% of patients, and is often resistant to standard therapies [3]. In LN, the prevalence of hypertension increases to 53.1% and correlates with the severity of renal involvement [4]. A hypertensive emergency (HE), a sudden and severe rise in blood pressure causing end-organ damage [5], occurs in approximately 7.6% of LN patients, typically in the context of elevated serum creatinine [6-8]. However, HE is rarely the initial manifestation of LN.

We report the case of a 64-year-old African American woman who presented with a hypertensive emergency as the first clinical indication of LN. While LN is more commonly diagnosed in younger women, this patient’s advanced age and normal baseline serum creatinine at presentation presented a diagnostic challenge. Additionally, minimal proteinuria obscured the renal origin of her severe hypertension. This case underscores an atypical presentation of LN in an older adult without overt renal dysfunction, highlighting the importance of thorough autoimmune and renal evaluations in patients with unexplained severe or treatment-resistant hypertension.

## Case presentation

The patient, a 64-year-old African American woman with a history of hypertension, heart failure with preserved ejection fraction, and non-alcoholic cirrhosis, presented to the emergency department with epistaxis. Hemostasis was achieved before arrival. On examination, her blood pressure was critically elevated at 221/127 mmHg, accompanied by sinus tachycardia at 110 beats per minute. She denied symptoms such as headache, visual disturbances, arthralgias, rashes, oral or nasal ulcers, hair loss, venous thromboembolism, or pregnancy loss. Her family history was negative for autoimmune diseases, and she reported no history of alcohol, recreational drug, or supplement use.

**Figure 1:**
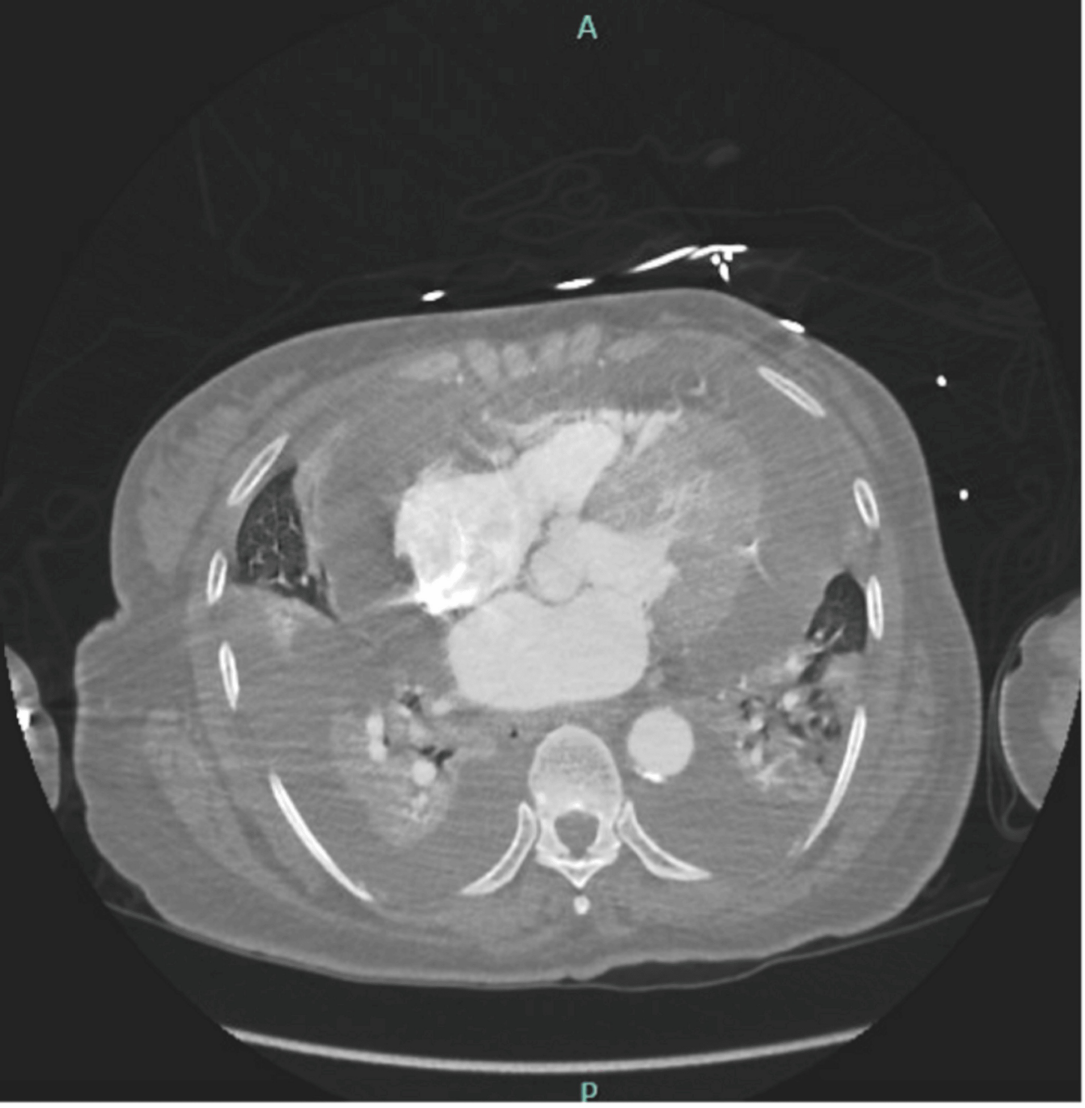
Computed tomography angiography of the chest showing bilateral pleural effusion and pericardial effusion.

Shortly after her initial evaluation, the patient developed acute hypoxia, with oxygen saturation dropping to the low 80s. Physical examination revealed 4+ pitting edema, increased respiratory effort, and bibasilar crackles. Initial laboratory tests showed mild elevations in liver enzymes, troponin, and B-type natriuretic peptide (Table 1). A chest radiography identified bilateral pleural effusions, while computed tomography angiography ruled out pulmonary embolism but confirmed pleural effusions and a large pericardial effusion (Figure 1). Pericardiocentesis was performed, yielding inflammatory effusion and negative for malignancy. The full kidney biopsy report report is in the Appendix.

**Table 1:** Pertinent lab results WBC: white blood cell count; AST: aspartate aminotransferase; ALT: alanine aminotransferase; ALP: alkaline phosphatase; BNP: B-type natriuretic peptide; UPCR: urine protein-to-creatinine ratio.

Hematology/Chemistry/Urine		Reference range
WBC (k/uL)	8.98	4.0–12.0
Hemoglobin(g/dL)	10.8	12.0–16.0
Platelet (k/uL)	219	150-440
Creatinine (mg/dL)	0.65	0.50–1.33
AST U/L	177	10–37
ALT (U/L)	51	12–78
ALP (U/L)	175	40–150
Troponin (Peak) (ng/mL)	0.09	<0.03
BNP (pg/mL)	1,465	<100
Urine Protein (mg/dL)	313.4	<14
Urine Creatinine (mg/dL)	111.96	Reference range not established
Urine WBC (none)/HPF	37	0/HPF
Urine RBC (none)/HPF	143	0/HPF
UPCR	2.8	<0.2

Given the unexplained hypertensive emergency, non-nephrotic range proteinuria, microscopic hematuria, and pericardial serositis, an extensive workup for autoimmune causes was initiated. Laboratory studies revealed positive antinuclear antibodies, anti-double-stranded DNA, anti-Smith, and anti-ribonucleoprotein antibodies (Table 2). Suspecting LN, we administered pulse-dose intravenous methylprednisolone (500 mg daily for three days), performed a kidney biopsy on day two, and received preliminary results within 24 hours.

**Table 2:** Autoimmune workup IgG: Immunoglobulin G, RNP: Ribonucleoprotein

Autoimmune Workup		Reference range
Antinuclear AB, S (ANA)	1:640, speckled pattern	<1:80
DNA Double-Stranded Ab, IgG, S (A-DNA)	>1000	<30.0
Smith Ab, IgG, S (Anti-Smith)	>8.0	<1.0
SS-A/Ro Ab, IgG, S	>8.0	<1.0
SS-B/La Ab, IgG, S	<0.2	<1.0
RNP Ab, IgG, S	>8.0	<1.0
Scl 70 Ab, IgG, S	<0.2	<1.0
Phospholipid Ab, IgG, S	<9.4	<15.0
Mitochondrial Ab, IgG, S	<0.1	<0.1
Jo 1 Ab, IgG, S	<0.2	<1.0
Complement C3 (mg/dL)	35	83-193
Complement C4 (mg/dL)	5	15-57

Kidney biopsy findings

**Figure 2:**
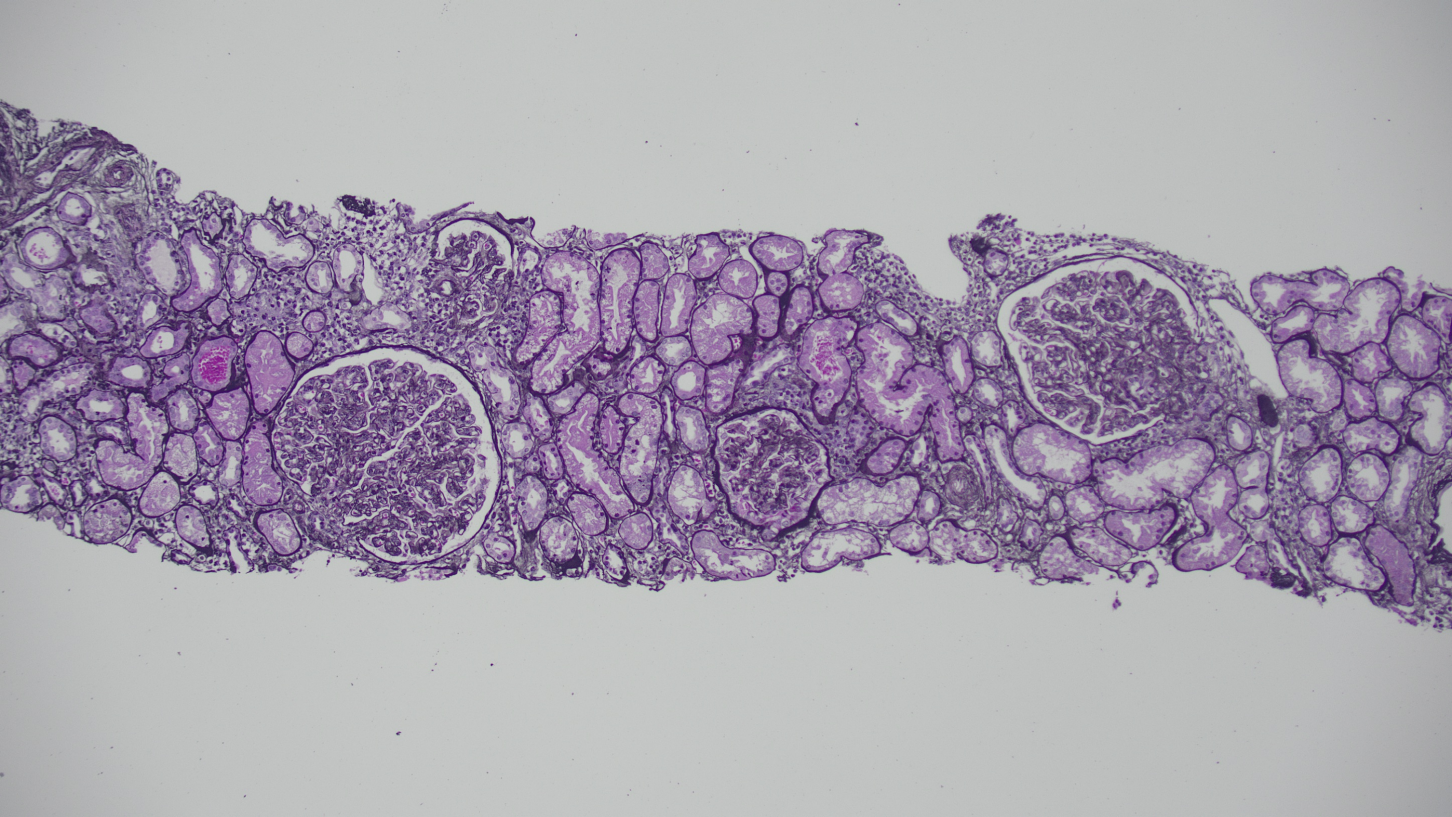
Endocapillary hypercellularity Jones stain

**Figure 3:**
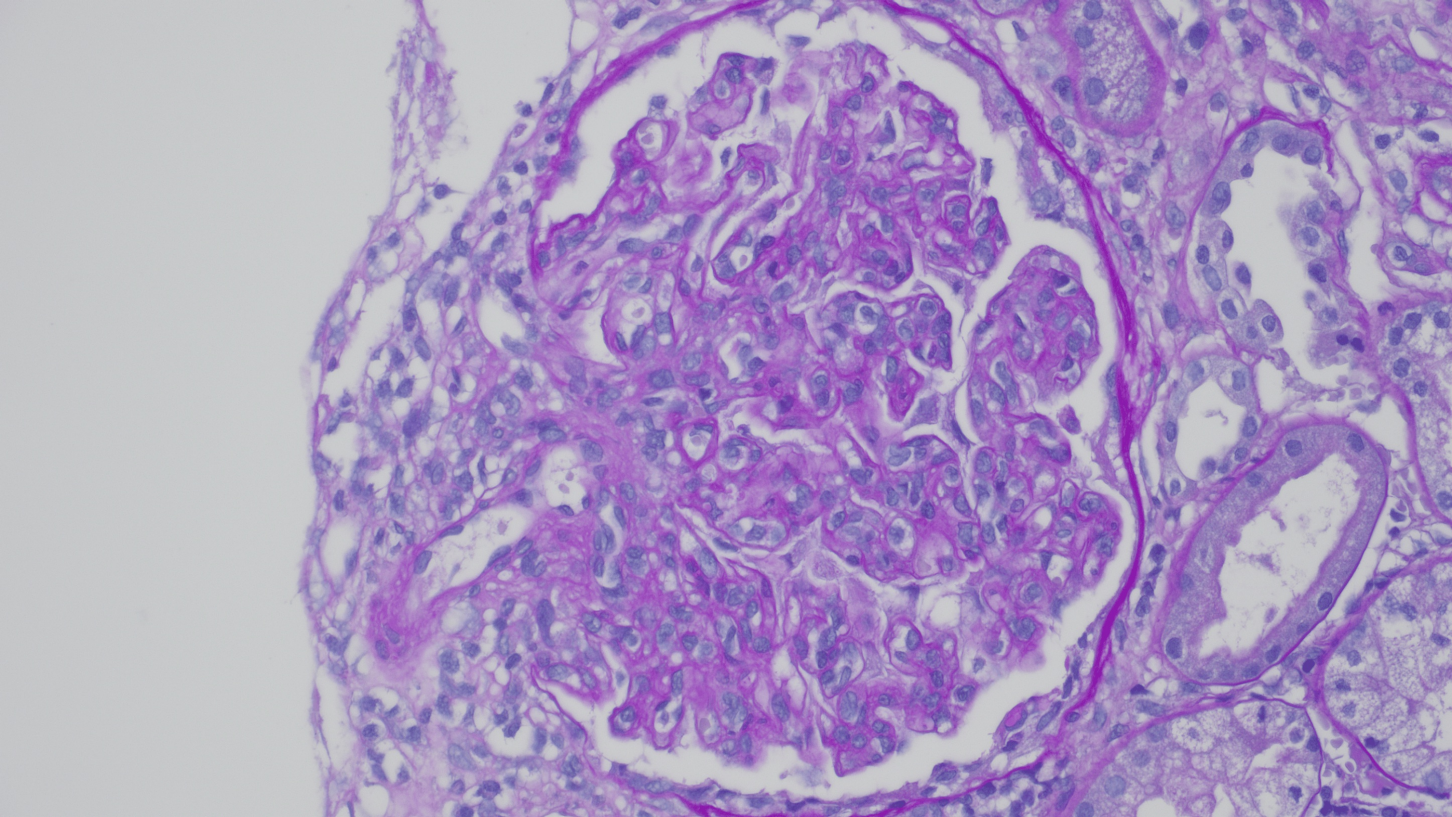
Endocapillary hypercellularity PAS stain

**Figure 4:**
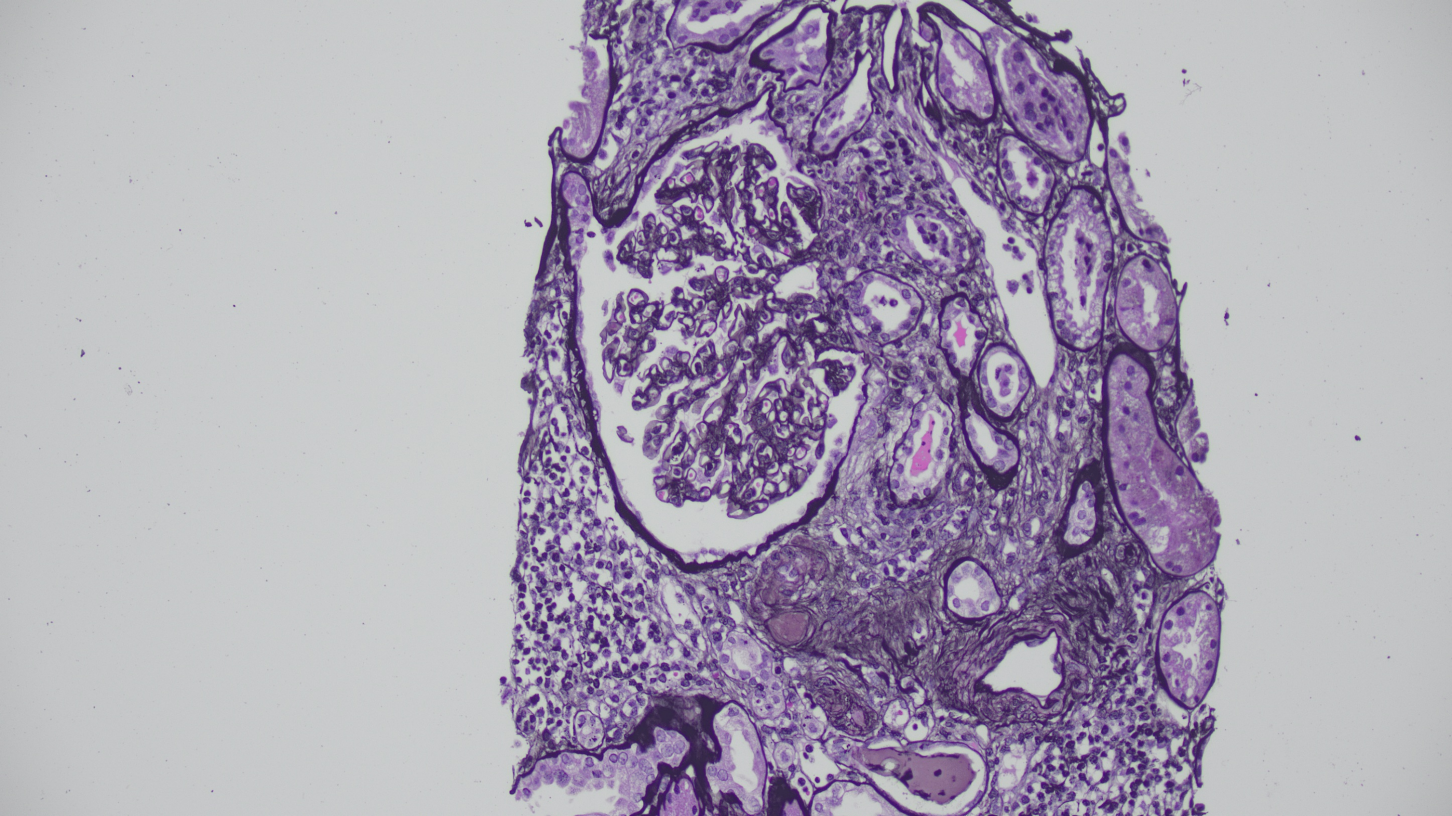
Thrombus in arteriole Jones stain

**Figure 5:**
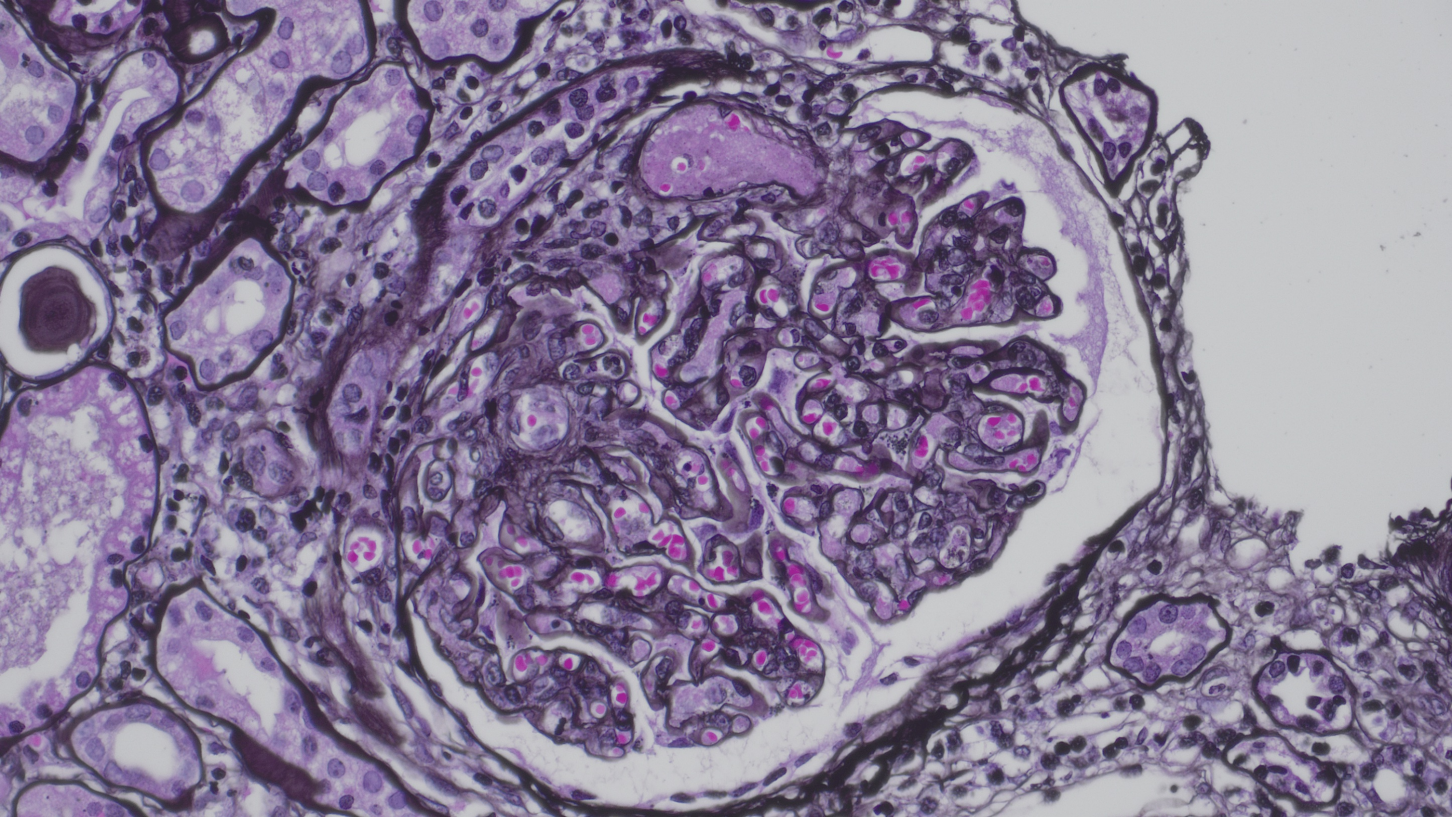
Thrombus in glomerulus and endocapillary hypercellularity double contours

Examination of 37 glomeruli revealed endocapillary hypercellularity in 20 glomeruli, with two exhibiting segmental adhesions. Two glomeruli show thrombi within the vascular pole with red blood cell fragments and fibrin occluding the lumen, and an additional arteriole shows an intraluminal fibrin thrombus. Immunofluorescence demonstrated full-house staining in mesangial areas and peripheral capillary loops, while electron microscopy confirmed mesangial and subendothelial deposits. These findings were consistent with diffuse proliferative LN, classified as Class IV (Figures 2-5).

After completing pulse-dose methylprednisolone, the patient was started on oral prednisone (60 mg daily), cyclophosphamide following the EuroLupus protocol (500 mg intravenous every two weeks for three months), and hydroxychloroquine (200 mg daily). By eight weeks, her creatinine briefly peaked at 1.34 mg/dL but returned to 0.6 mg/dL, while proteinuria improved from 2.8 g/day to 1-1.1 g/day, indicating partial remission. Blood pressure was well controlled with carvedilol, with plans to transition to angiotensin-converting enzyme (ACE) inhibitors or angiotensin receptor blockers (ARBs).

## Discussion

HE accounts for approximately 0.5% of emergency department visits and is associated with severe complications, including stroke, myocardial infarction, and renal failure. HE may arise from essential hypertension or secondary causes such as endocrine disorders, medications, or renovascular diseases. Renovascular causes include glomerulonephritis, scleroderma renal crisis, and renal artery stenosis [9]. Notably, a study evaluating 83 patients with HE identified only four cases attributable to SLE [10].

In SLE patients, hypertension is an emerging concern due to its strong association with cardiovascular and renal complications, as well as increased mortality, particularly in younger populations. Among women under 40 years of age, 40% of those with SLE had hypertension compared to 10% in the control group [11]. Hypertension and HE in SLE arise through mechanisms such as autoantibodies, inflammatory cytokines, corticosteroid use, and renal involvement [12]. Immune complex deposition in the kidneys, leading to LN, is the primary renal pathology in SLE [13]. Class IV LN, characterized by its diffuse proliferative nature, is most frequently associated with hypertension and HE [14].

Although LN is a common cause of hypertension in SLE, emerging evidence suggests that hypertension may occur independently of LN [4]. Secondary contributors to hypertension and HE, such as renal artery stenosis, vasculitis, or thrombotic microangiopathy, may also present concurrently [14]. Notably, HE can develop during the treatment course of SLE rather than solely at the time of initial diagnosis [8].

Timely and accurate diagnosis of lupus nephritis complicated by malignant hypertension is critical. Early initiation of immunosuppressive therapy, including corticosteroids, not only mitigates disease activity but also aids in blood pressure control and improves long-term renal outcomes [6].

It remains unclear whether current hypertension management guidelines are fully applicable to patients with SLE. Consequently, hypertension in SLE is typically managed according to standard protocols used for the general population [15]. However, ACEis and ARBs are particularly effective in lupus patients, especially those with nephritis. A retrospective review of 19 LN patients with malignant hypertension found that adding ACEis/ARBs to immunosuppressive therapy significantly improved blood pressure control and stabilized or enhanced renal function in most cases [6].

A distinctive feature of this case was the presence of pericardial effusion, an inflammatory form of serositis, accompanied by microscopic hematuria. These findings broadened the differential diagnosis and prompted further evaluation for an autoimmune etiology, ultimately leading to the diagnosis of lupus nephritis. Interestingly, the kidney biopsy revealed features suggestive of thrombotic microangiopathy, though serological confirmation was lacking.

While awaiting kidney biopsy results, the patient received intravenous methylprednisolone at 500 mg daily for three days, followed by oral prednisone at 1 mg/kg/day. Cyclophosphamide was initiated per the EuroLupus protocol [16]. The decision to use cyclophosphamide over mycophenolate mofetil was influenced by concerns regarding the patient’s adherence to long-term therapy.

A kidney biopsy revealed focal areas of thrombotic microangiopathy (TMA). Anticardiolipin antibodies were negative, and there was no evidence of hemolysis. Given these findings, we opted to continue immunosuppressive therapy and defer plasma exchange.

## Conclusions

This case underscores that a hypertensive emergency can serve as the initial manifestation of lupus nephritis, even in older patients with previously normal renal function. Recognizing subtle indicators such as proteinuria and serositis is crucial for timely diagnosis and management. A multidisciplinary approach is essential for early intervention, which can significantly improve outcomes in lupus nephritis.

## Appendices

Full kidney biopsy report

Comment

The biopsy included 40 glomeruli examined across all modalities, of which six were globally sclerosed and two showed segmental sclerosis. Diagnostic findings indicated diffuse lupus nephritis, classified as ISN/RPS class IV. Immunofluorescence revealed near full-house staining in mesangial regions and peripheral capillary loops, correlating with endocapillary hypercellularity in over half of the glomeruli. Electron microscopy confirmed mesangial and subendothelial deposits. Among the 37 glomeruli assessed in detail via light microscopy and toluidine blue scout sections for EM, 20 exhibited segmental-to-global endocapillary hypercellularity, including two with segmental sclerosis and adhesion. No evidence of necrosis or crescents was observed. Additionally, two glomeruli contained fibrin thrombi at the vascular pole, and one arteriole adjacent to another glomerulus displayed intraluminal thrombi, consistent with thrombotic microangiopathy. Immunofluorescence also identified focal tubular basement membrane deposits affecting approximately 10% of the tubular basement membranes, as well as bland vascular deposits, though corresponding changes were absent on light microscopy. Approximately 20% of tubules exhibited acute tubular injury with vacuolization and blebbing, while tubulointerstitial fibrosis affected about 20% of the tissue. These findings indicate mild chronicity and mild activity consistent with the features described above.

Microscopic description

Light Microscopy

Multiple sections of the biopsy were prepared, including three H&E, three PAS, and three Jones' silver-stained slides. The biopsy revealed four pieces of cortex and one corticomedullary junction, collectively containing 32 glomeruli, five of which were globally sclerosed in a nonspecific obsolescent pattern. The remaining glomeruli exhibited a mild increase in mesangial cells and matrix. Focal segmental spikes and occasional pinpoint holes were noted on the glomerular basement membrane in the silver stain, along with frequent but segmental double-contour appearances. No corrugation was observed. Two glomeruli demonstrated adhesion with segmental sclerosis.

There was no evidence of hyalinosis, crescents, or necrosis. Two glomeruli showed thrombi at the vascular pole, characterized by red blood cell fragments and fibrin occluding the lumen, while an additional arteriole displayed an intraluminal fibrin thrombus.

Fifteen glomeruli exhibited segmental endocapillary hypercellularity, two of which also showed segmental sclerosis and adhesion. Approximately 20% of the interstitial tissue displayed fibrosis, accompanied by proportional tubular atrophy, occasional proteinaceous casts, and rare red blood cell casts. No crystals or polarizable material were identified.

Mild interstitial lymphocytic infiltrates were observed in areas of scarring, without tubulitis. Approximately 20% of tubular epithelial cells showed vacuolization, with no evidence of PMNs or eosinophils. The vascular findings included thrombi as described above, with no vasculitis or necrosis in the vascular walls. Interlobular arteries exhibited moderate intimal fibrosis, while larger arteries were not sampled.

Immunofluorescence Microscopy

Duplicate frozen sections, each containing three glomeruli, were also analyzed. One glomerulus was nearly globally sclerosed, while the other two demonstrated increased cellularity without necrosis or crescents on hematoxylin and eosin-stained frozen sections. These sections were stained with antisera for IgG, IgA, IgM, C3, C1q, kappa, lambda, and polyvalent antisera. The findings included 3+ diffuse global granular mesangial and 2+ finely granular capillary loop staining for IgG and no glomerular staining for IgA. IgM exhibited 2+ diffuse global granular mesangial staining, while C3 and C1q displayed 2-3+ diffuse global granular mesangial and capillary wall staining. Polyvalent stains showed a 2+ diffuse global granular mesangial and 1-2+ segmental-to-global granular capillary loop staining, consistent with kappa and lambda (0 to 3+ scale).

Casts stained 2+ for IgA, polyvalent, kappa, and lambda. Arteries showed 1+ focal C3 and 2-3+ C1q in focal arterioles and arteries, along with 1+ polyvalent, kappa, and lambda staining. Approximately 10% of tubular basement membranes exhibited 1+ granular IgG and IgM, as well as 2+ C1q staining (0 to 3+ scale). No nuclear staining was detected, and negative controls were appropriately negative.

Electron Microscopy

One tissue sample was submitted in glutaraldehyde for electron microscopy. Scout sections revealed five glomeruli with segmental endocapillary hypercellularity but no necrosis or crescents. One of these glomeruli was selected for thin-section analysis. The glomerular basement membrane thickness, excluding areas with deposits, ranged from 513 to 780 nm (normal average in adult women: 325 nm). This finding may reflect changes associated with hypertension or early dysmetabolic injury. Subepithelial deposits were absent. Frequent small-to-medium subendothelial deposits were observed, accompanied by segmental cellular interposition. Approximately 50%-60% foot process effacement was noted. Segmental endocapillary hypercellularity was present, with occasional polymorphonuclear leukocytes in capillary lumens. Reticular aggregates and fibrin tactoids were not detected. There was a mild increase in mesangial matrix and a moderate increase in mesangial cells, with frequent small-to-medium mesangial deposits. Mild-to-moderate collagen accumulation was observed in the interstitium, while no deposits were identified along the tubular basement membranes in the limited electron microscopy sample.

## References

[1] Justiz VAA, Goyal A, Varacallo MA (2024). Systemic Lupus Erythematosus. https://pubmed.ncbi.nlm.nih.gov/30571026/.

[2] Aringer M, Costenbader K, Daikh D (2019). 2019 European League Against Rheumatism/American College of Rheumatology classification criteria for systemic lupus erythematosus. Ann Rheum Dis.

[3] Gandelman JS, Khan OA, Shuey MM (2020). Increased incidence of resistant hypertension in patients with systemic lupus erythematosus: a retrospective cohort study. Arthritis Care Res (Hoboken).

[4] Shaharir SS, Mustafar R, Mohd R (2015). Persistent hypertension in lupus nephritis and the associated risk factors. Clin Rheumatol.

[5] Alley WD, Schick MA (2024). Hypertensive Emergency. https://pubmed.ncbi.nlm.nih.gov/29261994/.

[6] Tao JL, Li H, Tang Y (2008). Lupus nephritis complicated with malignant hypertension: from renal vascular pathology to clinical relevance. Chin Med Sci J.

[7] Soliman AR, Hassan AA, Soliman MA (2017). Malignant hypertension in lupus nephritis. JSM Renal Med.

[8] Choe JY, Park SH, Kim JY (2010). A case of systemic lupus erythematosus presenting as malignant hypertension with hypertensive retinopathy. Korean J Intern Med.

[9] Siddiqi TJ, Usman MS, Rashid AM (2023). Clinical outcomes in hypertensive emergency: a systematic review and meta-analysis. J Am Heart Assoc.

[10] Yu SH, Whitworth JA, Kincaid-Smith PS (1986). Malignant hypertension: aetiology and outcome in 83 patients. Clin Exp Hypertens A.

[11] Sabio JM, Vargas-Hitos JA, Navarrete-Navarrete N (2011). Prevalence of and factors associated with hypertension in young and old women with systemic lupus erythematosus. J Rheumatol.

[12] Taylor EB, Ryan MJ (2016). Understanding mechanisms of hypertension in systemic lupus erythematosus. Ther Adv Cardiovasc Dis.

[13] Almaani S, Meara A, Rovin BH (2017). Update on lupus nephritis. Clin J Am Soc Nephrol.

[14] Baldwin DS, Gluck MC, Lowenstein J, Gallo GR (1977). Lupus nephritis. Clinical course as related to morphologic forms and their transitions. Am J Med.

[15] Tselios K, Koumaras C, Urowitz MB, Gladman DD (2014). Do current arterial hypertension treatment guidelines apply to systemic lupus erythematosus patients? a critical appraisal. Semin Arthritis Rheum.

[16] Houssiau FA, Vasconcelos C, D'Cruz D (2002). Immunosuppressive therapy in lupus nephritis: the Euro-Lupus Nephritis Trial, a randomized trial of low-dose versus high-dose intravenous cyclophosphamide. Arthritis Rheum.

